# Sertraline treatment prevents motor dysfunction in a Huntington's disease mouse model and functional decline in patients

**DOI:** 10.1016/j.neurot.2025.e00716

**Published:** 2025-08-06

**Authors:** Marta Garcia-Forn, Carla Castany-Pladevall, Jordi Creus-Muncunill, Arantxa Golbano, Geòrgia Escaramís, Jesús Pérez-Pérez, Uxue Balantzategi, Marina Hernan-Godoy, Verónica Brito, Jaime Kulisevsky, Eulàlia Martí, Esther Pérez-Navarro

**Affiliations:** a Facultat de Medicina i Ciències de la Salut, Institut de Neurociències, Universitat de Barcelona, Barcelona, Catalonia, Spain; b Institut d’Investigacions Biomèdiques August Pi i Sunyer (IDIBAPS), Barcelona, Catalonia, Spain; c Centro de Investigación Biomédica en Red sobre Enfermedades Neurodegenerativas (CIBERNED), Madrid, Spain; d Centro de Investigación CIBER Epidemiología y Salud Pública (CIBERESP), Barcelona, Spain; e Movement Disorders Unit, Department of Neurology, Hospital de la Santa Creu i Sant Pau, Biomedical Research Institute Sant Pau (IIB-Sant Pau), Barcelona, Spain

**Keywords:** 4E-BP1, Enroll-HD, Human fibroblasts, Puromycin, R6/1 mouse, Striatum

## Abstract

Molecular alterations underlying Huntington's disease (HD) are not fully elucidated and no curative therapies are available. We have described that increased translation efficiency participates in the motor symptoms in the R6/1 HD mouse model. Here, we evaluated whether sertraline, a widely used antidepressant drug, that modulates translation in cancer cells, could ameliorate motor and cognitive symptoms in HD. We also investigated if alterations in translation efficiency occur in fibroblasts from HD patients and serve as a possible biomarker. As an index of translation efficiency levels, we analyzed puromycin incorporation and phosphorylated 4E-BP1 levels in striatal primary cultures and striatum from R6/1 mice, and in HD patients' fibroblasts, with or without sertraline treatment. Motor learning and coordination were analyzed in treated mice by accelerating rotarod, balance beam and vertical pole tests. Clinical data from the Enroll-HD dataset were analyzed to evaluate the potential effects of sertraline treatment in the disease progression. We report that sertraline treatment: 1) modulates translation efficiency in striatal primary neurons expressing mutant huntingtin; 2) prevents motor dysfunction in R6/1 mice and normalizes translation efficiency in the striatum, and 3) delays the decline in functional performance of HD patients. Moreover, puromycin incorporation is increased in fibroblasts from HD patients in a CAG length-dependent manner and is modulated by sertraline treatment. Altogether, our results suggest sertraline as a promising candidate for HD clinical trials to slow down disease progression and that puromycin incorporation in fibroblasts could serve as a pharmacological biomarker for certain treatments.

## Graphical abstract

**Figure:**
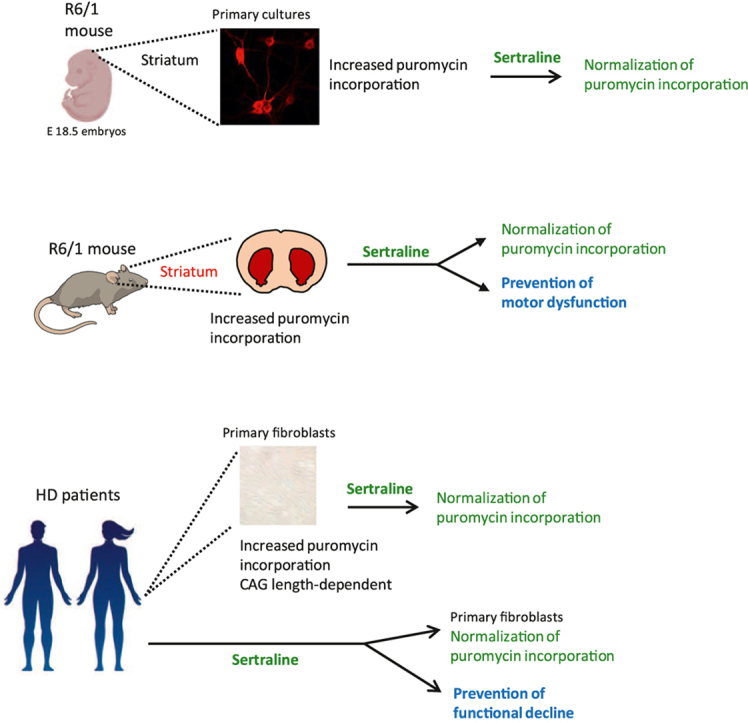

## Introduction

Huntington's disease (HD) is an autosomal dominant neurodegenerative disorder caused by an unstable expansion of the CAG repeat in the exon 1 of the huntingtin gene (*HTT*), which results in a mutated huntingtin protein (mHTT). The GABAergic medium-sized spiny neurons of the striatum are preferentially vulnerable to mHTT, and their dysfunction results in the characteristic motor disturbances of HD [1].

Molecular alterations underlying this disease are not fully elucidated. We have shown that increased translation efficiency in the striatum is one important pathogenic mechanism [2]. Initiation of translation is mainly regulated by the phosphorylation of the eukaryotic translation initiation factor 4E (eIF4E)-binding protein 1 (4E-BP1). In standard conditions, 4E-BP1 is bound to the cap-binding eukaryotic translation initiation factor 4E (eIF4E), avoiding it to form the eIF4F complex. When 4E-BP1 is phosphorylated at Thr37/Thr46, eIF4E is released and able to form the eIF4E complex, allowing the initiation of cap-dependent translation [3]. This phosphorylation is considered a limiting step [4] whose dysregulation, in addition to HD [2], has been detected in some other neurological disorders such as fragile X syndrome, autism and Parkinson's disease [[5], [6], [7]]. Proving the participation of this alteration in the pathophysiology of HD, intraventricular injection of 4EGI-1, an inhibitor of eIF4G-eIF4E interaction, normalized translation efficiency levels, and ameliorated motor symptoms and corticostriatal long-term depression deficits in the R6/1 HD mouse model [2].

In HD, although it has long been postulated that the age of onset inversely correlates with the number of CAG repeats, environmental and genetic factors may play a role as sources of variability, making it difficult to predict [8]. Indeed, a huge variability in the age of onset has been observed among individuals with the same number of CAG repeats [9]. Thus, identifying peripheral markers would be useful to predict the onset of symptoms in presymptomatic individuals, for monitoring disease progression in symptomatic patients and also to test the efficacy of emerging therapies. Since HTT is a ubiquitously expressed protein, evidence shows a broad toxic effect of mHTT also in the peripheral tissues suggesting that neurons and peripheral cells could share degeneration mechanisms. Indeed, peripheral tissues, such as saliva, fibroblasts and blood cells, reproduce alterations found in HD brains [10]. Although some of these alterations seemed promising, none of them has been implemented as a biomarker for regular use. In fact, it has been suggested that the combination of many biomarkers might be the most efficient tool for HD.

Here, we aim to analyze whether sertraline, a widely used antidepressant drug which modulates protein synthesis in cancer cells [11], could be a good therapeutic approach to ameliorate motor symptoms in HD by targeting the increased translation efficiency. We also aim to investigate if alteration in translation efficiency also occur in fibroblasts from HD patients and serve as a possible biomarker.

## Materials and Methods

### Striatal primary cultures

Primary cultures, obtained from E.18 wild-type and R6/1 mouse embryo [12], were produced individually from each embryo in a genotype blind manner. Tails were used for DNA extraction and presence/absence of m*Htt* was determined by PCR amplification as previously described [13]. Cells were seeded onto at a density of 80,000 ​cells/cm^2^ or 400,000 ​cells/cm^2^ for immunofluorescence and protein extraction, respectively.

### Human primary fibroblasts culture

Fibroblasts were derived from sterile, non-necrotic biopsy samples obtained from non-affected individuals and HD patients at different stages of the disease (Suppl. Table 1). Informed written consent was obtained from each subject before participation in the study, after the nature, purpose and risks of the study were explained. Biopsies were processed as described [14]. All fibroblast cell lines were maintained in DMEM with 25 ​mM glucose supplemented with 10 ​% v/v FBS, 1 ​% v/v penicillin/streptomycin and 1 ​% v/v amphotericin B.

### HD mouse model

Wild-type and R6/1 transgenic mice (B6CBA) expressing the exon-1 of m*Htt* with 115 CAG repeats were used. Mouse genotyping and CAG repeat length determination were performed as described [13]. Only male mice were used for the experiments as we have well-established timepoints for the assessment of the motor dysfunction and molecular alterations they display (see for example: [[15], [16], [17], [18]]). Mice were housed together in numerical birth order, with access to food and water *ad libitum* in a colony room kept at 19-22 ​°C and 40–60 ​% humidity, under a 12:12 light/dark cycle. Data were recorded for analysis by microchip mouse number. Procedures were carried out in accordance with the National Institutes of Health Guide for the Care and Use of Laboratory Animals, and approved by the local animal care committee of the Universitat de Barcelona (99/01), and Generalitat de Catalunya (99/1094).

### Sertraline treatment

Human fibroblasts and R6/1 mouse striatal primary cultures (DIV14) were treated with fresh medium containing either vehicle (DMSO) or 10 ​μM of sertraline for 4 ​h. For primary striatal cultures, B27 supplement was removed from the medium at DIV10 to avoid interferences with the treatment [19]. Wild-type and R6/1 mice, 12-week-old, received a daily intraperitoneally injection of vehicle (5 ​% Polysorbate 80 in H_2_O; Sigma; W291706) or sertraline (20 ​mg/kg; Toronto Research Chemicals) during 4 weeks.

### Behavioral analysis

Behavior was analyzed after 3 weeks of treatment by using the open field, the accelerating rotarod, the balance beam and the vertical pole as described [2]. During the testing days, sertraline was administered 1 ​h prior starting the test.

### Surface sensing of translation (SUnSET) method

In cultured cells, the medium was replaced with fresh medium containing 1 ​μM puromycin (Sigma-Aldrich; P8833) and incubated during 30 ​min. After incubation, medium was withdrawn and cells were processed for protein extraction and Western blot. Wild-type and R6/1 mice were killed by cervical dislocation 1 ​h after the last vehicle or sertraline injection. Brain was quickly removed and maintained in oxygenated artificial cerebrospinal fluid (ACSF; in mM: 125 NaCl, 2.5 KCl, 1.2 NaH_2_PO_4_, 1.2 MgCl_2_, 2.4 CaCl_2_, 26 NaHCO_3_, 11 glucose). Corticostriatal coronal slices (400 ​μm) were obtained using a conventional vibratome (Leica VT1200) and processed for the SUnSET method as described [20]. Slices were incubated during 1 ​h in oxygenated ACSF at 32 ​°C, and subsequently treated with puromycin (5 ​μg/ml) for 45 ​min. Then, striata were dissected out and flash frozen, and processed for protein extraction and Western blot. Puromycin incorporation was determined by measuring total lane smear signal from 250 to 25 ​kDa and normalizing against α-tubulin.

### Western blot analysis

Protein extraction and Western blot analysis was performed as described [21]. Membranes were incubated over night with primary antibodies (Suppl. Table 2), washed twice with Tris-buffered saline-tween 20 (0.1 ​%) after incubation, and incubated for 1 ​h at room temperature with the appropriated horseradish peroxidase conjugated secondary antibody (1:2000; Promega). The reaction was visualized with the Western Blotting Luminol Reagent (Santa Cruz Biotechnology; Santa Cruz, CA, USA). Western blot replicates were scanned and quantified using a computer-assisted densitometric analysis (Gel-Pro Analyzer, version 4; Media Cybernetics).

### Immunofluorescence

Cells were washed with phosphate-buffered saline (PBS) and fixed with 4 ​% paraphormaldehyde (PFA) in PBS for 10 ​min at room temperature. To block the action of PFA, cells were incubated with 0.2 ​M glycine for 20 ​min at room temperature. After quenching with 50 ​mM NH_4_Cl for 10 ​min, cells were permeabilized in blocking buffer containing 1 ​% BSA +0.2 ​% gelatin +0.2 ​% Triton X-100 in PBS at room temperature. After blocking, cells were incubated with anti-Puromycin (1:200; Sigma-Aldrich; MABE343) and anti-NeuN (1:200; Cell Signaling; 24307) for 30 ​min at room temperature. Next, cells were washed three consecutive times with PBS and incubated with Alexa Fluor 488 donkey anti-rabbit IgG and Cyanine 3 rabbit anti-mouse IgG (1:200; Jackson ImmunoResearch; 711-545-152 and 315-166-047). Nuclei were stained with DAPI Fluoromount.

### Enroll-HD database

Clinical data from the fifth Enroll-HD periodic dataset (PDS5) was analyzed to evaluate the potential beneficial effects of sertraline treatment in the disease progression of HD patients. Enroll-HD is a global multi-center longitudinal observational study, which results in a clinical research platform designed to facilitate clinical research in HD [22]. PDS5 contains clinical data from 21,116 Enroll-HD participants collected annually under local ethical approval. Only HD patients (both Pre-motor Manifest (PM) and Motor Manifest (M)) with a minimum of a 3-year follow-up were included in this study.

### Statistical analysis

For comparison between groups, Shapiro-Wilk test was used to assess normality. For normally distributed data (equal variance), Student's *t*-test was performed when comparing two groups and one- or two-way ANOVA for comparison between more than two groups. For not normally distributed data, Mann-Whitney and Kruskal-Wallis tests were used for comparison between two or more than two groups, respectively. All tests were followed by a post hoc test as indicated in the figure legends. A 95 ​% confidence interval was used and values of p ​< ​0.05 were considered as statistically significant. The Enroll-HD Database was analyzed by a Linear Mixed Effects Model (LMM) to evaluate differences in disease time evolution between groups of patients, two at a time, as it allows the control of the correlation structure of the data given by the fact that different measures of the same patient are collected over time.

## Results

### Translation efficiency is increased in R6/1 mice striatal primary neurons and is normalized by acute sertraline treatment

**Fig. 1:**
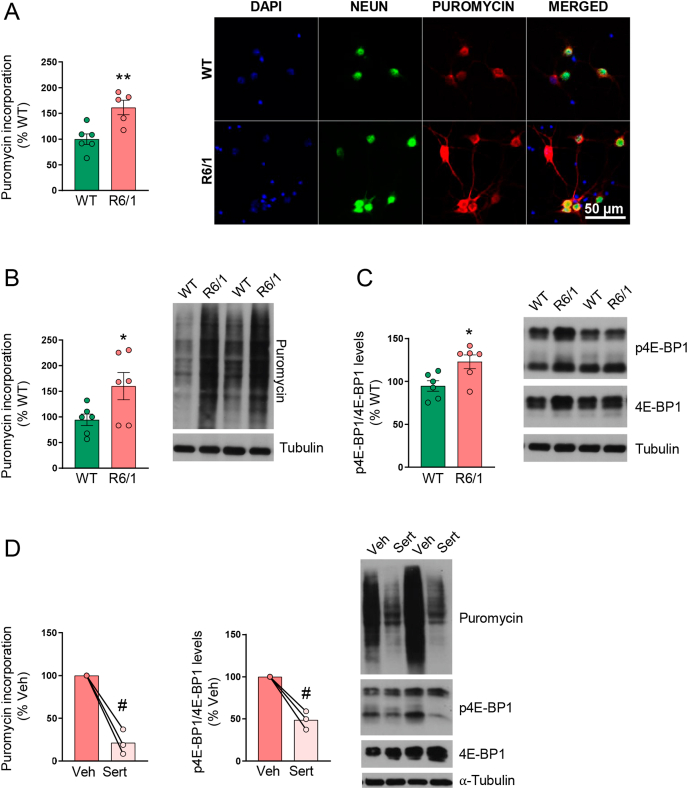
*Translation efficiency is increased in striatal R6/1 mouse primary cultures and sertraline treatment normalizes its levels*. (**A**) Puromycin incorporation was analyzed by immunocytochemistry and (**B**) Western blot, and (**C**) p4E-BP1 levels by Western blot, in striatal primary cultures obtained from wild-type (WT) and R6/1 mouse at DIV 14. In graphs, values are expressed as a percentage of WT cultures and shown as mean ​± ​SEM. (**D**) R6/1 mice striatal primary cultures were treated with vehicle (Veh; DMSO) or sertraline (Sert; 10 ​μM) at DIV 14 during 4 ​h. Puromycin incorporation and p4E-BP1 levels were analyzed by Western blot. In graphs, values are expressed as a percentage of vehicle treated cultures and shown as mean ​± ​SEM. Tubulin was used as loading control. Each point corresponds to the value from an individual culture. Representative immunoblots (**B**, **C**, **D**) and images (**A**) for each condition are shown. Unpaired (**A-C**) and two-tailed paired (**D**) Student's t-test, ∗p ​< ​0.05, ∗∗p ​< ​0.01 compared to WT mice; ^#^p ​< ​0.05, compared to vehicle-treated cultures.

Puromycin incorporation and p4E-BP1 protein levels were measured in striatal primary neurons derived from wild-type and R6/1 mice, at 14 DIV. In comparison to the wild-type ones, both parameters were increased in R6/1 mice striatal primary neurons (Fig. 1A–C). According to previous results showing that sertraline down-regulates translation initiation [11], addition of sertraline to wild-type (Suppl. Fig. 1) or R6/1 (Fig. 1D) mice striatal primary neurons decreased both puromycin incorporation and p4E-BP1 levels.

### Sertraline treatment prevents motor dysfunction in R6/1 mice and normalizes translation efficiency in the striatum

**Fig. 2:**
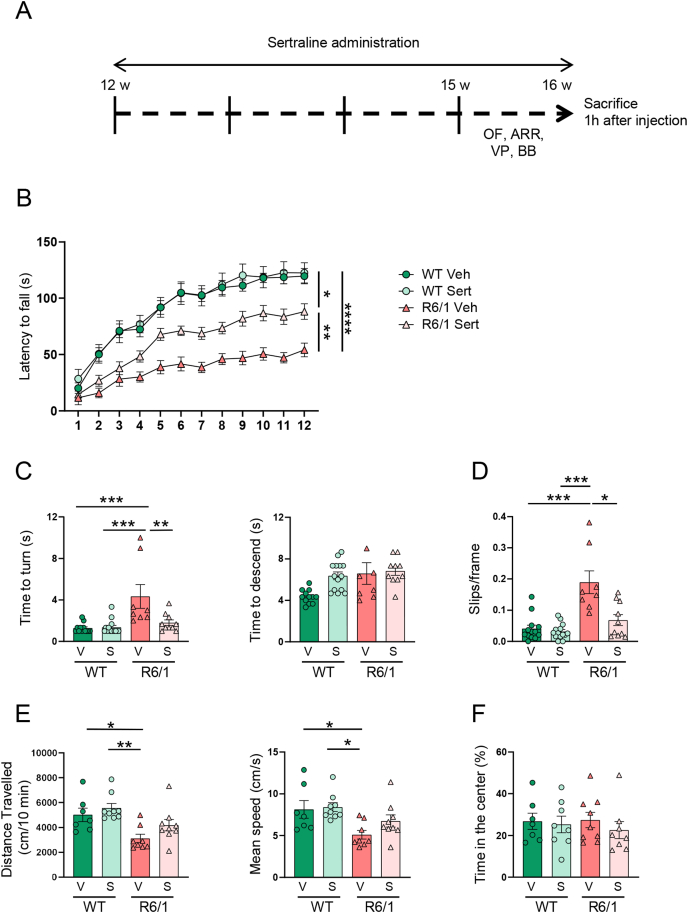
*Sertraline treatment prevents motor deficits in R6/1 mice*. (**A**) Schematic representation of the experimental design performed. Wild-type (WT) and R6/1 mice, at 12 weeks of age (w), were intraperitoneally injected with vehicle (Veh; H_2_O 5 ​% Polysorbate 80) or sertraline (Sert; 20 ​mg/kg) during 4 weeks. Behavioral tests were performed during the last week of treatment, except the open field (OF) that was also assessed before treatment. AR ​= ​accelerating rotarod test; BB ​= ​balance beam; VP ​= ​vertical pole. (**B**) Accelerating rotarod test was performed for four consecutive days (3 trials per day). The latency to fall per test and group is represented as mean ​± ​SEM (WT Veh and WT Sert, n ​= ​19; R6/1 Veh, n ​= ​14; R6/1 Sert, n ​= ​17). (**C**) Vertical pole: time (in seconds, s) to turn (left) and time to descend (right) were recorded after placing the mice upwards to the pole. Three trials were conducted. (**D**) Balance beam: Graph shows the number of slips committed per frame in 2 ​min. (**E**) Locomotor activity and (**F**) anxiety were measured in the open field. V: vehicle; S: Setraline. In all graphs, data are shown as mean ​± ​SEM. (**C-E**) Each point corresponds to the value from an individual mouse. Two-way ANOVA with Bonferroni's as a *post hoc* test, ∗p ​< ​0.05, ∗∗p ​< ​0.01; ∗∗∗p ​< ​0.001; ∗∗∗∗p ​< ​0.0001. Last trial statistical significances are shown in (**B**).

**Fig. 3:**
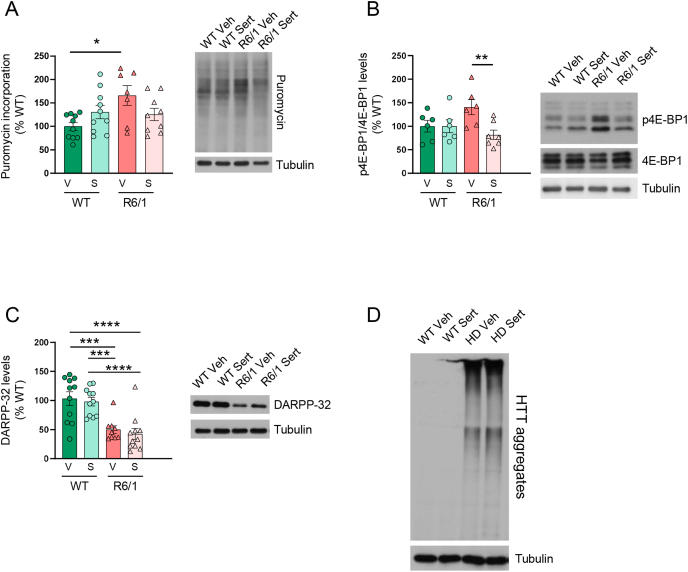
*Sertraline treatment normalizes protein synthesis rate in the striatum*. (**A**) Puromycin incorporation, (**B**) phospho-4E-BP1 (p4E-BP1) levels, (**C**) DARPP-32 protein levels and (**D**) mHTT protein levels (insoluble mHTT was detected in the stacking gel) were examined by Western blot in the striatum of wild-type (WT) and R6/1 mice treated with vehicle (V: Veh) or sertraline (S: Sert) at the end of treatment. Tubulin was used as loading control. Representative immunoblots are shown. In all graphs, values are expressed as a percentage of WT mice treated with vehicle and shown as mean ​± ​S.E.M. Each point corresponds to the value from an individual mouse. Two-way ANOVA with Bonferroni's as a *post hoc* test, ∗p ​< ​0.05, ∗∗p ​< ​0.01; ∗∗∗p ​< ​0.001; ∗∗∗∗p ​< ​0.0001.

Wild-type and R6/1 mice, 12-weeks-old, were treated with sertraline and motor behavior was analyzed beginning at 3 weeks of treatment (Fig. 2A). Sertraline treatment only exerted effects on R6/1 mice (Fig. 2B–F). It partially prevented the development of motor dysfunction, as shown in the rotarod and balance beam tests (Fig. 2B–D), and prevented the decrease in spontaneous locomotor activity (Fig. 2E). Moreover, it did not affect anxiety behavior in wild-type and R6/1 mice (Fig. 2F). Mice were killed 1 ​h after the last sertraline injection, and puromycin incorporation and p4E-BP1 protein levels were analyzed in the striatum. Although sertraline treatment decreased both puromycin incorporation and p4E-BP1 in striatal primary cultures from wild-type mice, the dose and time of treatment used did not exert effects in wild-type mice striatum (Fig. 3A and B). In contrast, R6/1 mice striatum showed normalized levels of puromycin incorporation and p4E-BP1 levels after sertraline treatment (Fig. 3A and B). However, sertraline treatment did not modify protein levels of DARPP-32 and insoluble forms of mHTT (Fig. 3C and D).

### Sertraline treatment slows down the functional performance decline in HD patients

Depression is one of the most prevalent psychiatric symptoms in HD and therefore, selective serotonin reuptake inhibitors (SSRIs), among other therapies, are often prescribed as symptomatic approaches [23]. Thus, we took advantage of the Enroll-HD database, the largest HD observational study conducted worldwide to analyze whether sertraline treatment, prescribed to HD patients as a symptomatic therapy for depression, might influence the disease progression course in terms of motor and functional outcomes.

**Fig. 4:**
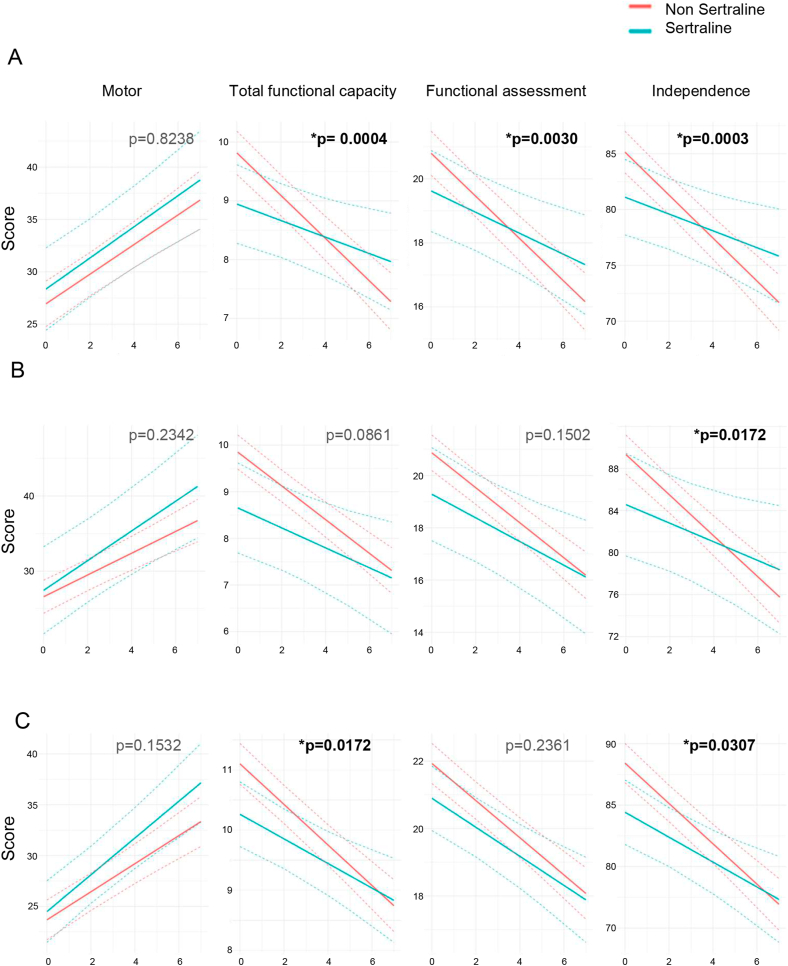
*Development of motor, total functional capacity, functional assessment and independence performance over seven years in HD patients.* (**A**) Comparison between HD patients treated with setraline±other antidepressants (n ​= ​63) and HD patients treated with other antidepressants (n ​= ​208). (**B**) Comparison between HD patients treated with sertraline as the unique antidepressant (n ​= ​30) and HD patients treated with other antidepressants (n ​= ​208). (**C**) Comparison between HD patients treated with sertraline±other antidepressants (n ​= ​63) and HD patients non-treated with anti-depressants (n ​= ​226). Statistical Analysis: Linear Mixed Effects Model (LMM) was applied to assess differences in disease time evolution between patient groups through the inclusion of time-group interaction term. The model also includes gender, age, CAG repeats, and HD status (PM or M) as potential confounders.

Only HD patients with a minimum of a 3-year follow-up were included in the study. We performed 3 different comparisons: (a) HD patients treated with sertraline with or without other antidepressants (n ​= ​63) and HD patients treated with other antidepressants but not sertraline (n ​= ​208); (b) HD patients treated with sertraline as the unique antidepressant (n ​= ​30) and HD patients treated with other antidepressants (n ​= ​208) and (c) HD patients treated with sertraline with or without other antidepressants (n ​= ​63) and HD patients non-treated with anti-depressants (n ​= ​226). As different measurements of the same patient are collected throughout time, from an annual clinic visit, LMM [24] was used to control the correlation structure of the data and evaluate differences in disease progression between these groups of patients. To assess the differences in disease time evolution between different patient groups, the model includes time as a linear effect and a time-group interaction term. This interaction term describes whether there are two different slopes of evolution depending on the groups, i.e. if the evolution of the disease over time is significantly different between the groups. Considering the slope rather than the mean difference between groups at each time point avoids the baseline mean effect since treated patients are already more severely affected at baseline. The model also includes gender, age, CAG repeats, and HD status (PM or M) as potential confounders. The response variables considered were: Total Motor Score (TMS) component of the Unified HD Rating Scale (UHDRS), used to evaluate the severity of motor symptoms; UHDRS-Total functional capacity (TFC), the functional assessment scale (FAS), and Independence Scale (IS), used to analyze functionality in HD patients. Results showed no significant differences on the motor function progression depending on the medication taken compared to the control group from the three comparisons described above. However, functionality performance was significantly better in the sertraline-treated groups. From the first comparison (a), we found that patients treated with sertraline presented significantly higher TFC, FAS and IS scores compared to the HD control group treated with other antidepressants (Fig. 4A). On the other hand, the second comparison showed that only the IS score was significantly higher in the sertraline-treated group (Fig. 4B). In the third comparison, sertraline treatment slowed the decline of the TFC and IS scores compared to the HD-control group non-treated with antidepressants (Fig. 4C). In summary, we identified group differences in terms of functionality scores in some treatment groups.

### Fibroblasts from HD patients expressing mHTT with less than 42 CAG repeats present increased translation

**Fig. 5:**
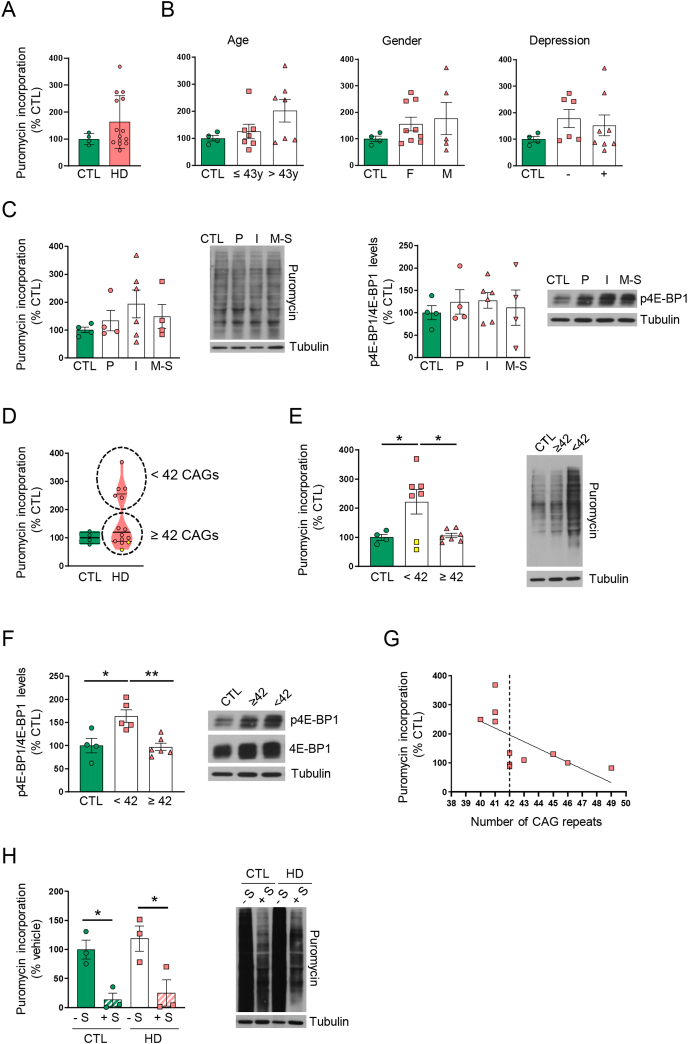
*Translation efficiency is increased in HD patients' fibroblasts expressing mHTT with less than* 42 CAG *repeats*. Puromycin incorporation, phospho-4E-BP1 (p4E-BP1) and 4E-BP1 levels were analyzed by Western blot in protein extracts obtained from non-affected individuals (CTL) and HD patients' primary fibroblast cultures. Tubulin was used as loading control. (**A**) Graph showing puromycin incorporation in fibroblasts from non-affected individuals and HD patients. (**B**) Puromycin incorporation was analyzed by Western blot and results were classified depending on age (y: years), gender and presence (+) or not (−) of depression. (**C**) Puromycin incorporation and p4E-BP1 levels analyzed by Western blot in human fibroblasts and classified depending on the disease stage (P: Pre-symptomatic; I: initial; M–S: Moderate-Severe). (**D**) Violin plot showing puromycin incorporation in CTL and in all the HD patients' fibroblasts analyzed. (**E**) Graph showing puromycin incorporation with values grouped according to the number of CAG repeats. (**F**) Graph showing phospho-4E-BP1 (p4E-BP1) and 4E-BP1 levels with values grouped according to the number of CAG repeats. (**G**) Graph showing the correlation between puromycin incorporation and the number of CAG repeats. (**H**) Puromycin incorporation in fibroblasts from CTL and HD patients after treatment with sertraline. In all graphs, results are expressed as a percentage of non-affected individuals and shown as mean ​± ​SEM. Each point corresponds to the value of an individual sample. Representative immunoblots are shown. Mann-Whitney test (**A**), one-way ANOVA with Bonferroni's as a *post hoc* test (**B** right and left panels, **C** right panel, **E**, **F**), Kruskal-Wallis with Dunn as a *post hoc* test (**B** middle panel, **C** and **H**), ∗p ​< ​0.05, ∗∗∗p ​< ​0.001, ∗∗∗∗p ​< ​0.0001. Yellow points in (**E**) indicate patients treated with sertaline.

We then asked whether increased translation could also be detected in more accessible cells and therefore serve as biomarkers of the disease progression and/or treatment efficacy. Puromycin incorporation and p4E-BP1 levels were analyzed in primary fibroblasts obtained from non-affected individuals and HD patients. We observed a trend towards increased levels of puromycin incorporation in fibroblasts from HD patients that did not reach statistical significance (Fig. 5A). We then classified the data depending on age, gender or the presence of depression. No differences were observed in puromycin incorporation (Fig. 5B). Similarly, no differences were observed in puromycin incorporation and p4E-BP1 when data was grouped by the disease stage (Fig. 5C). We then analyzed whether the number of CAG repeats could be a determinant of puromycin incorporation. When results were plotted in a violin plot, there were two groups within the HD group that were clearly separated (Fig. 5D). Fibroblasts showing increased levels of puromycin incorporation (Fig. 5D and E) corresponded to HD patients expressing mHTT with less than 42 CAG repeats, except two values (yellow points in Fig. 5D and E). Fibroblasts showing increased puromycin incorporation also showed increased levels of p4E-BP1 (Fig. 5F), indicating that translation is only increased in a subset of patients in a CAG-dependent manner (Fig. 5G). Interestingly, when analyzing the characteristics of the patients expressing mHTT with less than 42 CAG repeats not showing increased puromycin incorporation in the fibroblasts (yellow points in Fig. 5D and E), we detected that they were being treated with sertraline, thus suggesting that sertraline would be normalizing translation. To study this possibility, we treated with sertraline control and HD patients’ fibroblasts. As shown in Fig. 5H, treatment with sertraline decreased puromycin incorporation.

## Discussion

Present results show that sertraline treatment modulates p4E-BP1 and puromycin incorporation levels in cells expressing mHTT both *in vitro* and *in vivo*. In addition, it prevents the development of motor dysfunction in R6/1 mice suggesting that some of the beneficial effects are mediated by translation modulation in the striatum. Moreover, sertraline treatment slows down the functional performance decline in HD patients but, unfortunately, it does not modulate motor function. Finally, puromycin incorporation is increased in HD patients’ fibroblasts in a CAG-dependent manner, an effect that is modulated by sertraline treatment.

Our previous study showed that translation efficiency is increased in HD striatal neurons and that its pharmacological normalization prevents R6/1 mice motor dysfunction [2]. However, the compound used does not cross the blood brain barrier. Therefore, one of our goals was to determine whether an FDA-approved drug already used to treat other brain pathologies, and shown to regulate translation, could modulate motor learning and coordination in a HD mouse model. Sertraline was a good candidate since it modulates translation in cancer cells [11], improves motor performance in R6/2 and N171-82Q HD mouse models [25,26], and is used to treat depression in HD patients [23]. First, we determined if sertraline could modulate translation in striatal neurons expressing mHTT. As observed previously in the striatum of HD mouse models [2], we found that puromycin incorporation and p4E-BP1 levels were increased in cultured striatal neurons from R6/1 mouse, an alteration that was modulated by sertraline treatment.

In sertraline-treated R6/1 mice, we observed a prevention of the decline in motor learning and coordination. Previous studies have already shown the effect of sertraline on motor learning in other HD mouse models that were attributed to an enhancement in neurogenesis and BDNF levels [25,26]. Here, we extend these results by showing that sertraline treatment also prevents motor coordination dysfunction. In correlation with this prevention, puromycin incorporation and p4E-BP1 levels were normalized in sertraline-treated R6/1 mice striatum. Since we have previously shown that inhibition of translation initiation in R6/1 mice brain prevents motor dysfunction [2], it is tempting to speculate that beneficial effects of sertraline are mediated, at least in part, by modulation of translation efficiency. Thus, sertraline treatment could exert its effects through the modulation of the serotoninergic system and the associated induction of BDNF [27,28], as described for other selective SSRIs, and also by correcting protein synthesis rate. The fact that treatment with other SSRIs, such as fluoxetine, failed to prevent motor dysfunction in R6/1 mice although increased neurogenesis [29] strengthens the suggestion that sertraline exerts its effects on motor function through other molecular mechanisms, namely regulation of translation efficiency. Moreover, other compounds showing beneficial effects in HD mouse models such as metformin [30,31], fingolimod [32,33], and misoprostol [34] regulate translation efficiency directly or indirectly [6,[35], [36], [37]]. Interestingly, the use of metformin in HD patients has been shown to associate with better cognitive function [38].

Since sertraline is used to treat depression in HD patients [23], we analyzed whether beneficial effects observed in R6/1 mice motor function could translate to patients. The Enroll-HD database [22] allowed us to analyze longitudinal data from a remarkable number of patients recruited at multiple sites worldwide. The strengths of the data presented here consist of the statistical model used, LMM, which allowed us to avoid the limitations coming from the baseline differences and potential confounders while focusing on the slope of evolution rather than the mean difference between groups at each time point [24]. Interestingly, we observed that sertraline treatment, alone or in combination with other antidepressants, delays the decline in functional performance with a consistent effect on IS. Unfortunately, we were unable to identify either positive or negative effects of sertraline on TMF, which can be related to the low number of samples or to the dose of sertraline administrated since doses used in mouse models are higher than doses used in humans [39]. Similarly, a previous study analyzing Enroll-HD database shows that treatment with tricyclic antidepressants does not have any effect on motor function but, in contrast to our results, they do not exert effects on functional outcomes [40]. Moreover, another study showed that motor-manifest HD patients treated with antidepressants, without considering the type of antidepressant, showed worse outcomes for TMS and TFC [41]. The effect of sertraline on TFC and FAS seemed to fade in patients prescribed with sertraline as the only antidepressant, which points to a synergistic effect rather than the sum of the effects of the individual drugs. In agreement, it has been reported that SSRIs and bupropion outperformed serotonin and norepinephrine reuptake inhibitors in alleviating depression in HD [42]. This, however, remains mere speculation since the lack of significance in the effect of sertraline in this group of patients could be explained by the relatively small sample size (n ​= ​30).

TFC decline in HD patients has been associated with whole brain, caudate and grey-matter atrophy [43]. It is difficult to indicate whether the effects of sertraline on TFC are due to the prevention of these brain alterations or to indirect effects by improving depressive symptoms or irritability that can also affect cognitive function. Although some studies point to a relation between depression and worse TFC [44,45], others indicate that depression alone does not impact on TFC [[46], [47], [48], [49]], whereas the presence of other neuropsychiatric symptoms, such as apathy and perseverative behavior, relate to decreased functional capacity [45,50]. Moreover, use of antidepressants has not been associated with TFC change [47] or insufficient data are available to draw conclusions [51]. Taken together, the effect of sertraline on the functional outcome of HD patients seems to be independent of its effects on depressive symptoms, and that it could be a better option than other antidepressants, at least to ameliorate the decrease in TFC, FAS and IS. Finally, we cannot determine whether the effect of sertraline on functional outcomes is due to its impact on translation efficiency or serotonin reuptake. However, it is clear that sertraline treatment regulates puromycin incorporation in fibroblasts from HD patients who inherited mHTT with less than 42 CAG repeats. It would be interesting to analyze whether the effect of sertraline on TFC, FAS and IS, is higher in those patients expressing mHTT with less than 42 CAG repeats than in the rest of HD patients, and whether HD patients that do not respond to sertraline are those carrying mHTT with more than 42 CAG repeats. Moreover, even if sertraline proves effective only in a subset of HD patients, this would not preclude its therapeutic potential. Given that sertraline is already prescribed to HD patients to treat depression, it would be feasible to use it for systematic evaluation of its efficacy.

We also show that puromycin incorporation is increased in HD patients’ fibroblasts in a CAG length-dependent manner, as alterations were only detected in fibroblasts derived from HD patients who inherited mHTT with less than 42 CAG repeats. In agreement, it has been previously shown that CAG repeat length impacts cognitive development in children carrying mHTT [52], alternative splicing defects [53] and lamin B1 levels [54] in fibroblasts derived from HD patients, and the ATP/ADP ratio in HD patient-derived lymphoblastoid cell lines [55]. Moreover, HTT and mHTT have been shown to interact with proteins that regulate translation [56,57] and maybe this interaction could vary depending on the length of the polyQ tract. Interestingly, soluble oligomers of polyQ-expanded HTT exon-1 (64Q) have been shown to interact with proteins that regulate translation, an interaction that does not occur when the polyQ is over 150 [58]. It is important to point that cell-type dependent somatic CAG expansion has been involved in HD pathogenesis [59,60]. In this sense, only the information about the inherited CAG repeat length is available for the present samples, as we did not analyze the number of CAG repeats for each of the human fibroblasts cell lines. Thus, we do not know the exact length of the polyQ tail of each sample, but it is unlikely that somatic instability is influencing our results as this phenomenon is typically slow in human fibroblasts and may require long-term culture to observe significant changes [61]. Unfortunately, due to the small number of samples, we were not able to classify puromycin incorporation depending on the disease stage in those patients expressing mHTT with less than 42 CAG repeats to know if alterations occur at pre-symptomatic stages and could serve as a biomarker. Importantly, we did not observe differences in puromycin incorporation nor in p4E-BP1 levels in fibroblasts of HD patients when classified depending on gender, suggesting that, at least in human cells, these molecular markers are not significantly influenced by sex. This would support our observations on the effect of sertraline in mice to possibly be true in both male and female patients. Overall, our results prove that analysis of puromycin incorporation in fibroblasts from HD patients expressing mHTT with less than 42 CAG repeats can be used to monitor pharmacological treatments known to modulate translation efficiency.

In summary, we show that sertraline modulates translation efficiency in cells expressing mHTT *in vitro* and *in vivo*. In the R6/1 HD mouse model, treatment with sertraline prevents the development of motor dysfunction and normalizes puromycin incorporation and p4E-BP1 levels in the striatum. Moreover, sertraline exerts a neuroprotective effect in HD patients in terms of functionality. Finally, puromycin incorporation is increased in fibroblasts from HD patients who inherited mHTT with less than 42 CAG repeats, an alteration that is modulated by sertraline treatment. Overall, sertraline arises as a promising candidate for HD clinical trials to slow down disease progression.

## Author contributions

Conceptualization and interpretation of the results, M.G.-F., C.C.-P. and E.P.-N.; methodology, M.G.-F., C.C.-P., J.C.-M., A.G., U.B., M.H., and V.B.; analysis of the results and preparing the figures, M.G.-F., C.C.-P. and E.P.-N.; analysis and interpretation of the Enroll-HD database, G.E. and E.M.; HD patient's follow-up, J.P.-P.; management of obtaining human skin biopsies, J.P–P. and J.K.; writing-original draft preparation, M.G.-F., E.P.-N.; writing-review and editing, all authors; supervision, E.P.-N. All authors have read and agreed to the published version of the manuscript.

## Declaration of competing interest

The authors declare that they have no known competing financial interests or personal relationships that could have appeared to influence the work reported in this paper.
