# Supplementary material for: Sertraline treatment prevents motor dysfunction in a Huntington's disease mouse model and functional decline in patients
Source: Neurotherapeutics. 2025 Aug 6;22(6):e00716. doi: 10.1016/j.neurot.2025.e00716 (PMC12664457; doi:10.1016/j.neurot.2025.e00716)
Supplement: Multimedia component 1 [file mmc1.pdf]

Supplementary Table 1 *Details of human fibroblasts*

ID	Gender	Age (years)	CAG repeats	Stage	Depression
001	Female	62	-	0	N
002	Female	41	-	0	N
003	Male	26	-	0	N
004	Female	45	-	0	N
005	Female	69	42	1	N
006	Female	56	41	1	N
007	Female	40	42	1	Y
008	Female	41	43	1	N
009	Male	43	41	2	N
010	Male	59	41	2	Y
011	Female	40	49	2	Y
012	Female	69	40	2	N
013	Male	43	41	2	Y
014	Female	39	45	2	Y
015	Female	70	41	3	Y
016	Male	59	46	3	N
017	Female	43	42	3	Y
018	Male	62	41	3	Y

Information about the gender, age, CAG repeat length (-, no information available), stage of disease (0, control; 1, pre-symptomatic; 2, initial; 3, moderated-advanced), and presence of depression (N, no depression; Y, depression)
