# Supplementary material for: Sertraline treatment prevents motor dysfunction in a Huntington's disease mouse model and functional decline in patients
Source: Neurotherapeutics. 2025 Aug 6;22(6):e00716. doi: 10.1016/j.neurot.2025.e00716 (PMC12664457; doi:10.1016/j.neurot.2025.e00716)
Supplement: Multimedia component 2 [file mmc2.pdf]

TABLE S2 Primary antibodies used for the Western blot

Antigen	Host	Dilution	Source	Identifier
Puromycin	Mouse	1:1000	Sigma-Aldrich	MAB343
Anti-4E-BP1	Rabbit	1:1000	Cell Signaling	9452
Anti-p4E-BP1 (Thr37/46)	Rabbit	1:1000	Cell Signaling	9459
DARPP-32 (clone 15)	Mouse	1:1000	BD Bioscience	611520
Huntingtin (EM48)	Mouse	1:1000	Millipore	MAB5374
Actin	Mouse	1:20000	MP Biochemicals	691000
α-Tubulin	Mouse	1:50000	Sigma Chemical Co.	6074
