# Supplementary material for: Sertraline treatment prevents motor dysfunction in a Huntington's disease mouse model and functional decline in patients
Source: Neurotherapeutics. 2025 Aug 6;22(6):e00716. doi: 10.1016/j.neurot.2025.e00716 (PMC12664457; doi:10.1016/j.neurot.2025.e00716)
Supplement: Multimedia component 3 [file mmc3.pdf]

Supplementary Figure 1

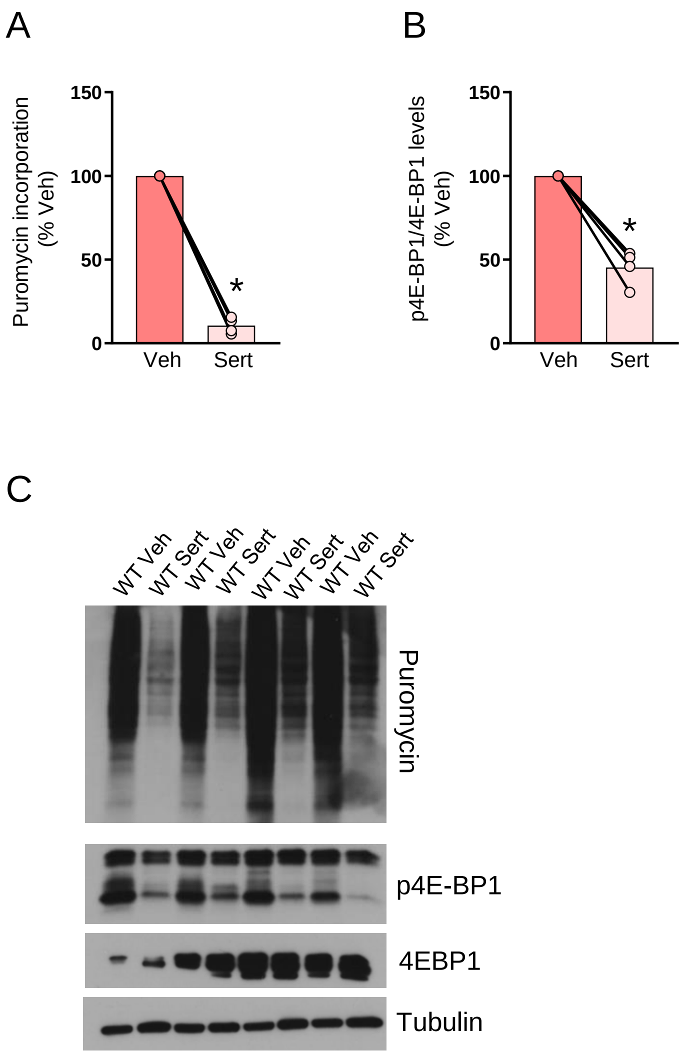

Suppl. Fig. 1. *Setraline treatment decreases puromycin incorporation and p4E-BP1 levels in wild-type mouse striatal primary cultures*. At DIV 14, wild-type mice striatal primary cultures were treated with vehicle (Veh; DMSO) or sertraline (Sert; 10µM) during 4 h. Puromycin incorporation (**A**) and p4E-BP1 (**B**) protein levels were analyzed by Western blot. Values are expressed as a percentage of vehicle treated cultures. Tubulin was used as loading control. (**C**) Uncropped blots are shown. Each point corresponds to the value from an individual culture. Student's t-test, *p<0.001 compared to vehicle-treated cultures.
